# Uncoupling Protein 2 Increases Susceptibility to Lipopolysaccharide-Induced Acute Lung Injury in Mice

**DOI:** 10.1155/2016/9154230

**Published:** 2016-02-07

**Authors:** Qin Wang, Jianchun Wang, Mingdong Hu, Yu Yang, Liang Guo, Jing Xu, Chuanjiang Lei, Yan Jiao, JianCheng Xu

**Affiliations:** Department of Pneumology, Xinqiao Hospital, The Third Military Medical University, No. 183, Xinqiao Main Street, Shapingba District, Chongqing 400037, China

## Abstract

Uncoupling protein 2 (UCP2) is upregulated in patients with systemic inflammation and infection, but its functional role is unclear. We up- or downregulated UCP2 expression using UCP2 recombinant adenovirus or the UCP2 inhibitor, genipin, in lungs of mice, and investigated the mechanisms of UCP2 in ALI. UCP2 overexpression in mouse lungs increased LPS-induced pathological changes, lung permeability, lung inflammation, and lowered survival rates. Furthermore, ATP levels and mitochondrial membrane potential were decreased, while reactive oxygen species production was increased. Additionally, mitogen-activated protein kinases (MAPKs) activity was elevated, which increased the sensitivity to LPS-induced apoptosis and inflammation. LPS-induced apoptosis and release of inflammatory factors were alleviated by pretreatment of the Jun N-terminal kinase (JNK) inhibitor SP600125 or the p38 MAPK inhibitor SB203580, but not by the extracellular signal-regulated kinase (ERK) inhibitor PD98059 in UCP2-overexpressing mice. On the other hand, LPS-induced alveolar epithelial cell death and inflammation were attenuated by genipin. In conclusion, UCP2 increased susceptibility to LPS-induced cell death and pulmonary inflammation, most likely via ATP depletion and activation of MAPK signaling following ALI in mice.

## 1. Introduction

Acute lung injury (ALI) and acute respiratory distress syndrome (ARDS) are common and major causes of acute respiratory failure accompanied by high mortality and morbidity [1]. Although decades of research brought considerable progress to understanding ALI/ARDS pathogenesis, the mortality of ALI and ARDS still remains high (30–40%) [2].

ALI/ARDS development is the result of uncontrolled inflammatory responses in the lungs, which involve neutrophil accumulation, diffuse endothelium and epithelial damage, air-blood barrier disruption, and the subsequent infiltration of peripheral inflammatory cells into lung tissues. This leads to the upregulation of inflammatory cytokines that induce lung edema, which ultimately results in tissue injury and severe immunopathology [3, 4]. Mitochondria are considered an important factor in alveolar epithelial damage. The mitochondria are involved in numerous apoptosis signaling pathways, such as regulating reactive oxygen species (ROS) production, adenosine triphosphate (ATP) balance, stabilizing mitochondrial membrane potential, or controlling calcium homeostasis [5–7]. Moreover, prior studies suggested that the intersection of mitochondrial biogenesis and inflammatory responses are important in disease [8]. However, the interaction of mitochondrial dysfunction and inflammatory response and their roles in the pathogenesis of ALI are not clear.

Uncoupling proteins (UCPs), members of the anionic proton transporter family, are located in the mitochondrial inner membrane, pumping protons from the inner membrane into the matrix to uncouple electron transport from ATP synthesis [9]. Uncoupling protein 2 (UCP2) contributes to a reduction in ATP production, inhibition of ROS, and stabilization of mitochondrial calcium balance and mitochondrial membrane potential [10]. The pathological function of UCP2 was found to be tissue and disease specific. UCP2 protects vascular cells [11] and brain cells [12] from oxidative stress. On the contrary, some studies have shown that UCP2 may negatively affect cellular function in some diseases, such as type 2 diabetes mellitus, insulin resistance [13], and acute liver injury [14]. UCP2 overexpression aggravated hypoxia/reoxygenation-induced ATP decline and loss of cellular viability in cardiomyocytes [15]. The mechanistic role of UCP2 in cell death is also contradictory. Although mild mitochondrial uncoupling by UCP2 may prevent cell death through attenuation of ROS production [16], UCP2 may facilitate apoptotic or necrotic cell death through ATP depletion [9].

Prior studies found that expression of UCP2 was increased in patients with systemic inflammation and infection [17], while the functional role of UCP2 in LPS-induced lung injury is still unclear. In the present study, we explored the association between pulmonary inflammation and UCP2 expression. Our findings indicated that UCP2 enhanced mitochondrial dysfunction and activated MAPK signaling, which increased susceptibility to LPS-induced alveolar epithelial cell death and inflammation in the lung, suggesting that UCP2 potentially contributes to disease progression of LPS-induced ALI in mice.

## 2. Experimental Procedures

### 2.1. Animals

Six 8-week-old adult male C57BL/6 mice, 20–25 g body weight, were purchased from the Laboratory Animal Center of the Third Military Medical University (Chongqing, China). The mice were housed in a specific pathogen-free facility and given free access to food and water. Mice were handled according to the National Institutes of Health Guidelines on the Use of Laboratory Animals. The study protocol was approved by the Animal Ethics Committee of the Third Military Medical University.

### 2.2. Adenovirus Gene Delivery

The recombinant adenovirus containing the mouse UCP2 gene was purchased from Genechem Company (Genechem Biotech Co., Shanghai, China). The adenovirus expressing no transgene was used as negative control (UCP2-NC). In order to avoid pulmonary inflammation caused by high doses of adenoviral vectors, C57BL/6 mice were anesthetized using sodium pentobarbital, and then gradient doses of UCP2-Ad (low-dose: 1 × 10^8^ plaque-forming units (pfu)/mouse, medium-dose: 5 × 10^8^ pfu/mouse, and high-dose: 1 × 10^9^ pfu/mouse) were administered* via* intranasal instillation as previously described [18]. Control mice were treated with either sterile saline or control adenovirus (UCP-NC) (5 × 10^8^ pfu). In the following studies, we used 5 × 10^8^ pfu/mouse UCP2-Ad to overexpress UCP2 in the airways of C57BL/6 mice, unless stated otherwise.

### 2.3. ALI Model

To establish the ALI model, mice were intraperitoneally injected with LPS (*Escherichia coli* 055:B5; Sigma, St. Louis, MO, USA) at a dose of 15 mg/kg body weight [19]. UCP2-Ad (5 × 10^8^ pfu/mouse) was administered three days prior to LPS administration. Genipin (Wako, Osaka, Japan) was injected by gavage 1 h before LPS administration at a dose of 100 mg/kg body weight [20]. To inhibit MAPK pathways, SB203580 (25 mg/kg), SP600125 (30 mg/kg), and PD98059 (30 mg/kg) were administered via intraperitoneal injection before LPS treatment. After 24 h, mice were sacrificed and the lungs were lavaged twice with 0.8 mL sterile saline each time to obtain bronchoalveolar lavage fluid (BALF). The BALF was centrifuged (700 ×g) for 10 min and supernatants were collected and frozen at −80°C for protein and cytokine enzyme-linked immunosorbent assay (ELISA) analysis. The BALF cells were resuspended in 1 mL phosphate buffered saline (PBS) and counted using a hemocytometer. Whole lung tissue without lavage was collected and stored at −80°C for Western blot analysis.

### 2.4. Pulmonary Edema Level

To calculate wet-to-dry (*W*/*D*) ratio, lung tissues from mice were weighed before and after being dried in an oven at 80°C for 48 h [21].

### 2.5. Lung Histopathology

Lung tissue that was not lavaged and was fixed in a 10% formalin solution overnight. Paraffin-embedded lungs were cut into 4 *μ*m sections. Tissue sections were stained with haematoxylin and eosin (H&E). Pathological changes were assessed quantitatively using a lung injury score as previously described [22]. Briefly, hyaline membranes, intra-alveolar edema, hemorrhage, epithelial necrosis, and recruitment of inflammatory cells on each section were assessed. The grading of severity was scored: grade 0 (normal); grade 1 (mild); grade 2 (moderate); grade 3 (severe). Grading severity was accumulated to achieve the lung injury score.

### 2.6. Immunohistochemical Analysis

Expression of UCP2 in lung sections was detected with an antibody specific to mouse UCP2 (Abcam Cambridge, MA, USA), as previously described [23]. Briefly, tissue sections were incubated for 1 h with 3% hydrogen peroxide (H_2_O_2_) to block endogenous peroxidase and then incubated with 5% normal goat serum for 30 min. Next, the sections were incubated at 4°C overnight with antibody against UCP2 (dilution 1 : 50). After washing, a horseradish peroxidase- (HRP-) labeled polymer antibody (Santa Cruz Biotechnology, CA, USA) was used as a secondary antibody. Diaminobenzidine (DAB) was used as the substrate, and sections were counterstained with haematoxylin.

### 2.7. TUNEL Staining

Apoptotic cells in sections (4 *μ*m) were detected by the terminal deoxynucleotidyl transferase-mediated dUTP nick end labeling (TUNEL) technique, using the* in situ* Cell Death Detection Kit (Roche Molecular Biochemicals, Mannheim, Germany). For quantitative cell death measurements, TUNEL positive cells and total cells were counted to calculate percentage of TUNEL positive pneumocytes over total cells in random fields (400x).

### 2.8. Measurement of Mitochondrial Membrane Potential, ATP Synthesis, and ROS Production

Mitochondria from mouse lungs were isolated and purified as described [24]. The mitochondrial ATP synthesis was measured with a luciferase/luciferin-based approach and ROS production was evaluated with a malondialdehyde (MDA) assay kit (Beyotime Institute of Biotechnology, Beijing, China). The mitochondrial membrane potential (Δ*ψ*m) was estimated using Rhodamine 123 (Sigma) as described [25].

### 2.9. Western Blot Analysis

Protein expression in lung tissues was evaluated by Western blot analysis as described previously [26]. Briefly, total protein extracts from lung tissue were separated by polyacrylamide gradient gels (8–15%; Invitrogen Corp., Carlsbad, CA, USA) and transferred onto polyvinylidene fluoride membranes (Amersham Pharmacia Biotech, Piscataway, NJ, USA). The membranes were incubated overnight with the primary antibodies anti-UCP2 (Abcam), anti-cytochrome c, anti-Bcl-2, anti-Bax, anti-caspase-3, anti-cleaved-caspase-3, anti-JNK, anti-p38, anti-p-JNK, or anti-p-p38 (all from Santa Cruz Biotechnology, CA, USA) followed by incubation with appropriate HRP-conjugated secondary antibodies (Sigma) for 2 h. Immunoreactive proteins were detected with an enhanced chemiluminescence system (ECL; Pierce Biotechnology, Rockford, IL, USA).

### 2.10. BALF Analysis

Proinflammatory cytokines including tumor necrosis factor- (TNF-) *α* and interleukins- (IL-) 1*β* and IL-6 in supernatants of BALF were measured using an ELISA reagent kit (Neobioscience Technology Company, Shenzhen, China). Total protein in BALF was assayed using the BCA Protein Assay Reagent Kit (Pierce, USA). The total cell number in BALF was counted on a hemocytometer.

### 2.11. Statistical Analysis

Data were expressed as means ± SEM. Student's *t*-test was used to compare two groups, and differences among multiple groups were analysed using ANOVA. Statistical analyses were performed using the SPSS 13.0 software and *P* < 0.05 was considered statistically significant.

## 3. Results

### 3.1. Up- or Downregulation of UCP2 Expression by UCP2 Recombinant Adenovirus or Genipin Treatment in Lungs of Mice

**Figure 1:**
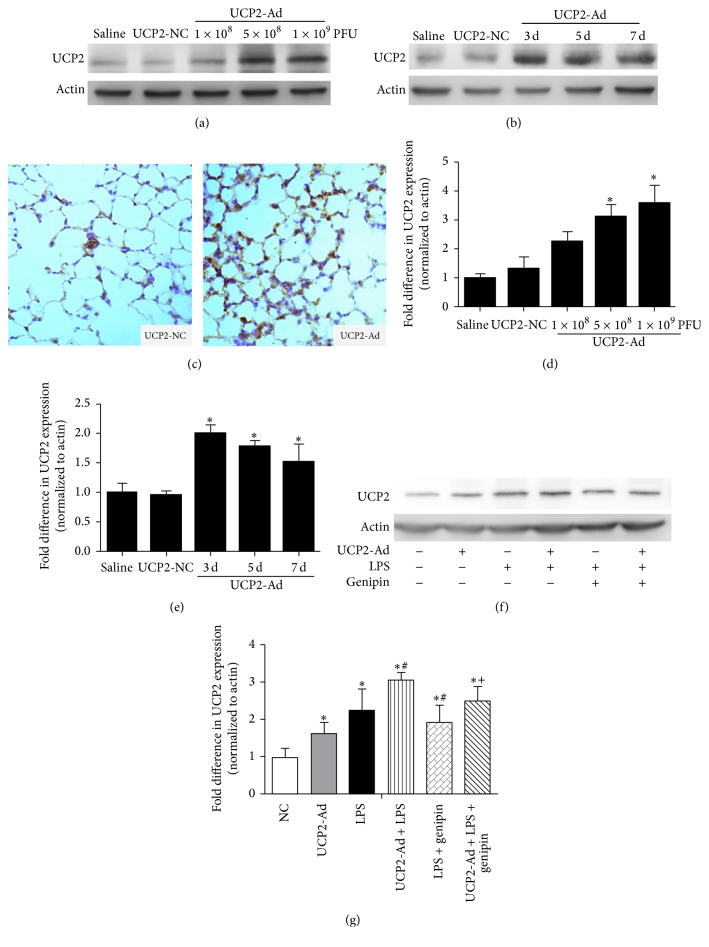
Regulation of UCP2 expression by UCP2 recombinant adenovirus or genipin in mouse lungs. (a) Western blot analysis detected UCP2 expression in lung homogenates of mice after intranasal administration of saline, UCP2-NC, or different UCP2-Ad virus doses (1 × 10^8^, 5 × 10^8^, or 1 × 10^9^ pfu/mouse). (b) Western blot analysis detected UCP2 expression in lung at 3, 5, and 7 days after administration of saline, UCP2-NC, or UCP2-Ad virus at a dose of 5 × 10^8^ pfu/mouse. (c) Immunohistochemical analysis of UCP2 overexpression with an antibody specific to mouse UCP2 (brown color) in the control group treated with UCP2-NC virus (control) and the UCP2-Ad-treated group (400x). (d, e) Densitometric analysis of UCP2 immunoreactive bands is shown as fold difference in UCP2 expression normalized to actin expression (*n* = 3, ^*∗*^ *P* < 0.05* versus* the saline group (control)). (f, g) Western blot analysis detected the role of genipin in UCP2 expression in lung tissue (*n* = 3, ^*∗*^ *P* < 0.05* versus* the saline group, ^#^ *P* < 0.05* versus* the LPS group, ^+^ *P* < 0.05* versus* the UCP2-Ad + LPS group). Values are presented as means ± SEM.

To determine the role of UCP2 in LPS-induced ALI, the expression of UCP2 was up- or downregulated by UCP2 recombinant adenovirus (Figures 1(a)–1(c)) or genipin treatment (Figure 1(f)), respectively. As shown in Figure 1(a), a 2–4-fold increase of UCP2 expression was observed after administration of increasing doses of UCP2-Ad compared to saline control levels. No significant difference of UCP2 expression between high-dose UCP2-Ad (1 × 10^9^ pfu/mouse) and medium-dose UCP2-Ad (5 × 10^8^ pfu/mouse) groups was found (Figure 1(d)). Therefore, a medium-dose of 5 × 10^8^ pfu/mouse was used in the following studies to induce UCP2 overexpression without triggering an inflammatory response. The increase of UCP2 expression was the highest at 3 days compared to 5 and 7 days after 5 × 10^8^ pfu/mouse UCP-Ad treatment (Figures 1(b) and 1(e)). These results confirmed that UCP2 overexpression in mouse lung can be accomplished with this adenovector-based technique, and elevated expression levels were maintained for the duration of the experiment, which was confirmed by immunohistochemistry (IHC) (Figure 1(c)). On the other hand, a pharmacological inhibitor of UCP [27], genipin, was used to downregulate the expression of UCP2 in mouse lungs. Genipin significantly suppressed the expression of UCP2 following LPS or UCP2-Ad treatment (Figures 1(f) and 1(g)).

### 3.2. UCP2 Promotes LPS-Induced Lung Injury* In Vivo*

**Figure 2:**
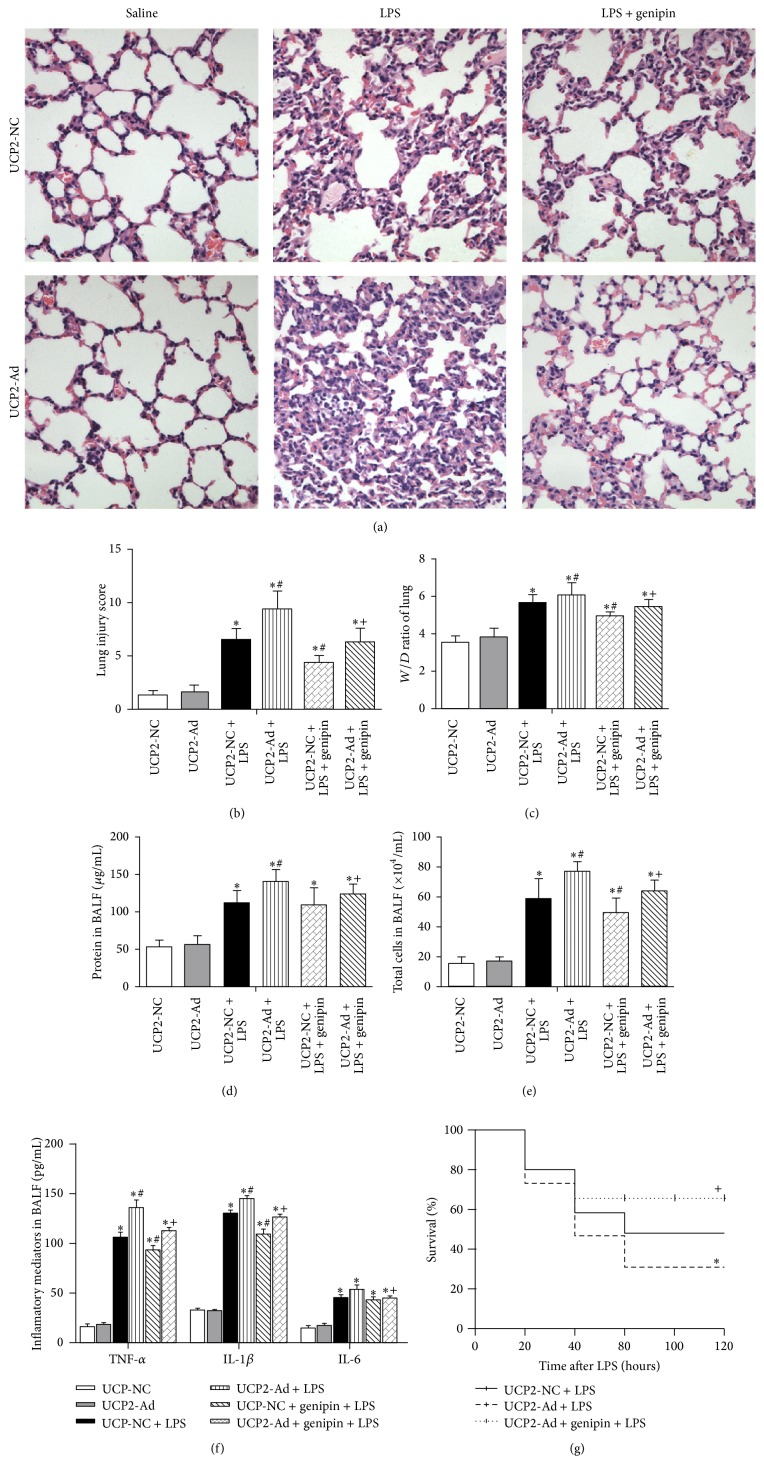
Effects of UCP2 on LPS-induced inflammatory lung injury, pulmonary edema, and survival rate. Mice were pretreated with UCP2-Ad, control virus (UCP2-NC), or genipin following injected LPS or saline. (a) Lungs from each group were processed for histological examination after H&E staining (400x). (b) Lung injury score in each group (*n* = 5). (c) The *W*/*D* ratio (*n* = 5). (d) Total protein concentrations in BALF. (e) Total cells number in BALF. (f) The levels of TNF-*α*, IL-1*β*, and IL-6 in the BALF were quantified by ELISA (*n* = 5). (g) The comparisons of survival rate in each group (*n* = 16). ^*∗*^ *P* < 0.05* versus* UCP2-NC + saline group, ^#^ *P* < 0.05* versus* UCP2-NC + LPS group, and ^+^ *P* < 0.05* versus* the UCP2-Ad + LPS group. Values are presented as means ± SEM.

To assess whether UCP2 plays a role in LPS-induced ALI, UCP2-NC, UCP2-Ad, or genipin treated groups were intraperitoneally injected with saline or LPS, and alterations in the lungs of the different groups were assessed by H&E staining (Figure 2(a)). Compared to untreated mice, no significant difference in lung tissue damage was found between UCP2-NC and UCP2-Ad groups (data not shown). UCP2 overexpression dramatically aggravated LPS-induced lung pathological injury (inflammatory cell infiltration as determined by H&E staining [22], edema of the alveolar septa, and alveolar hemorrhage), whereas genipin inhibited lung damage (Figure 2(b)). Additionally, UCP2-Ad administration dramatically increased the *W*/*D* ratio (Figure 2(c)) and protein amount (Figure 2(d)) in BALF after LPS treatment, indicating that UCP2-Ad aggravated alveolar-capillary barrier damage in ALI. Moreover, UCP2 administration increased the total number of BALF cells (Figure 2(e)) and TNF-*α* and IL-1*β* (Figure 2(f)) in BALF after LPS challenge. Despite the established trend of increased IL-6 in the UCP2-Ad + LPS group, there was no statistically significant difference between the UCP2-NC + LPS and the UCP2-Ad + LPS groups (Figure 2(f)). More importantly, the survival rate in the UCP2-Ad + LPS group was significantly reduced compared to the UCP2-NC + LPS group (Figure 2(g)). Downregulation of UCP2 protected UCP2-overexpressing mice from LPS-induced alveolar-capillary barrier damage (Figures 2(c) and 2(d)) and from proinflammatory cytokine release (Figure 2(f)) and increased the survival rate in mice (Figure 2(g)). Taken together, these results suggest that UCP2 overexpression* in vivo* enhanced LPS-induced lung inflammation and injury in mice. Downregulation of UCP2 protected mice from LPS-induced lung injury.

### 3.3. UCP2 Enhances Mitochondrial Dysfunction in LPS-Induced ALI

**Figure 3:**
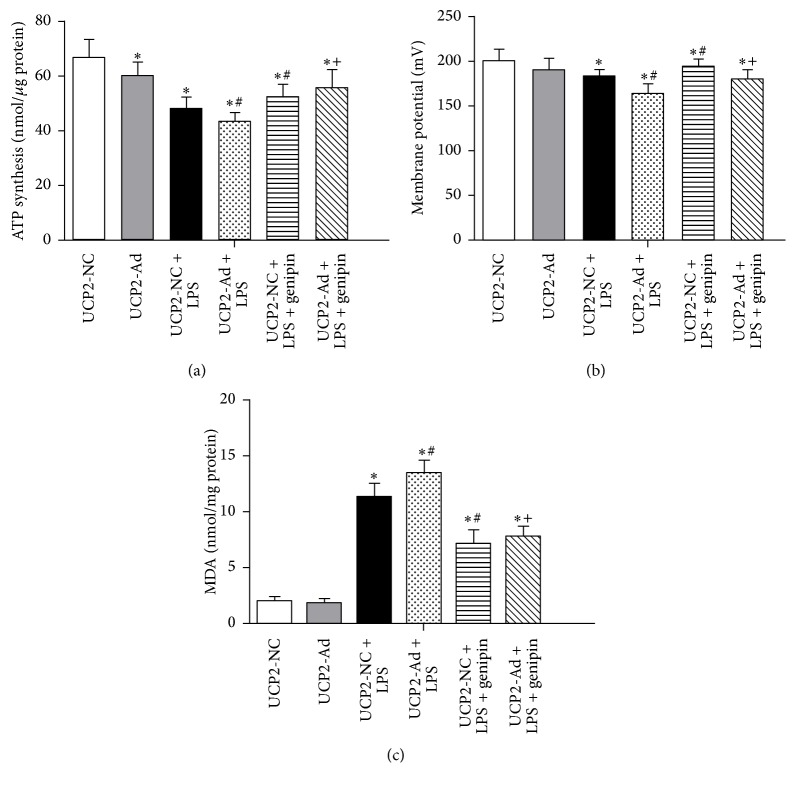
Effects of UCP2 on mitochondrial ATP levels, membrane potential, and ROS production in lung tissue. (a) ATP levels were measured by a luciferase-based assay in isolated mitochondria. (b) The membrane potential (Δ*ψ*m) of isolated mitochondria (1 mg/mL) was measured using Rhodamine 123 (1 mM). (c) Lipid peroxidation (MDA assay) were measured in the lung tissues of mice (^*∗*^ *P* < 0.05* versus* UCP2-NC + saline group, ^#^ *P* < 0.05* versus* UCP2-NC + LPS group, and ^+^ *P* < 0.05* versus* UCP2-Ad + LPS group). Values are presented as means ± SEM (*n* = 8).

It has been previously shown that mitochondrial functions including ATP synthesis, ROS production, and mitochondrial membrane potential are altered in lung mitochondria from mice and rats with ALI [6, 7]. It is thought that these alterations of mitochondrial functions play a decisive role in cell death progression. Therefore, mitochondrial bioenergetics were examined to determine the effect of UCP2 in LPS-induced injury (Figure 3). As shown in Figure 3(a), mitochondrial ATP synthesis rates in UCP2-NC and UCP2-Ad groups were of no significant difference (*P* > 0.05) but significantly declined after LPS treatment. ATP synthesis rates in the UCP2-Ad + LPS group were significantly decreased compared to the UCP2-NC + LPS group. Genipin pretreatment inhibited LPS- or UCP2-induced ATP depletion (Figure 3(a)). Additionally, when the effect of UCP2 on mitochondrial membrane potential (Δ*ψ*m) was measured, we found that UCP2-Ad induced a decrease in Δ*ψ*m compared to UCP2-NC treated mice after LPS administration, while genipin inhibited the decrease of Δ*ψ*m induced by UCP2 (Figure 3(b)). Lipid peroxidation, a readout for ROS production, was enhanced in the LPS-treated groups compared to the saline control groups and was higher in the UCP2-Ad + LPS group compared to the UCP2-NC + LPS group (Figure 3(c)). Moreover, genipin inhibited LPS-induced ROS production (Figure 3(c)). In conclusion, these results support the notion that UCP2 induces mild mitochondrial uncoupling, leading to a decrease in mitochondrial ATP levels and membrane potential, but enhancement of ROS production in LPS-induced ALI in mice.

### 3.4. UCP2 Promotes Alveolar Apoptosis in LPS-Induced ALI in Mice

**Figure 4:**
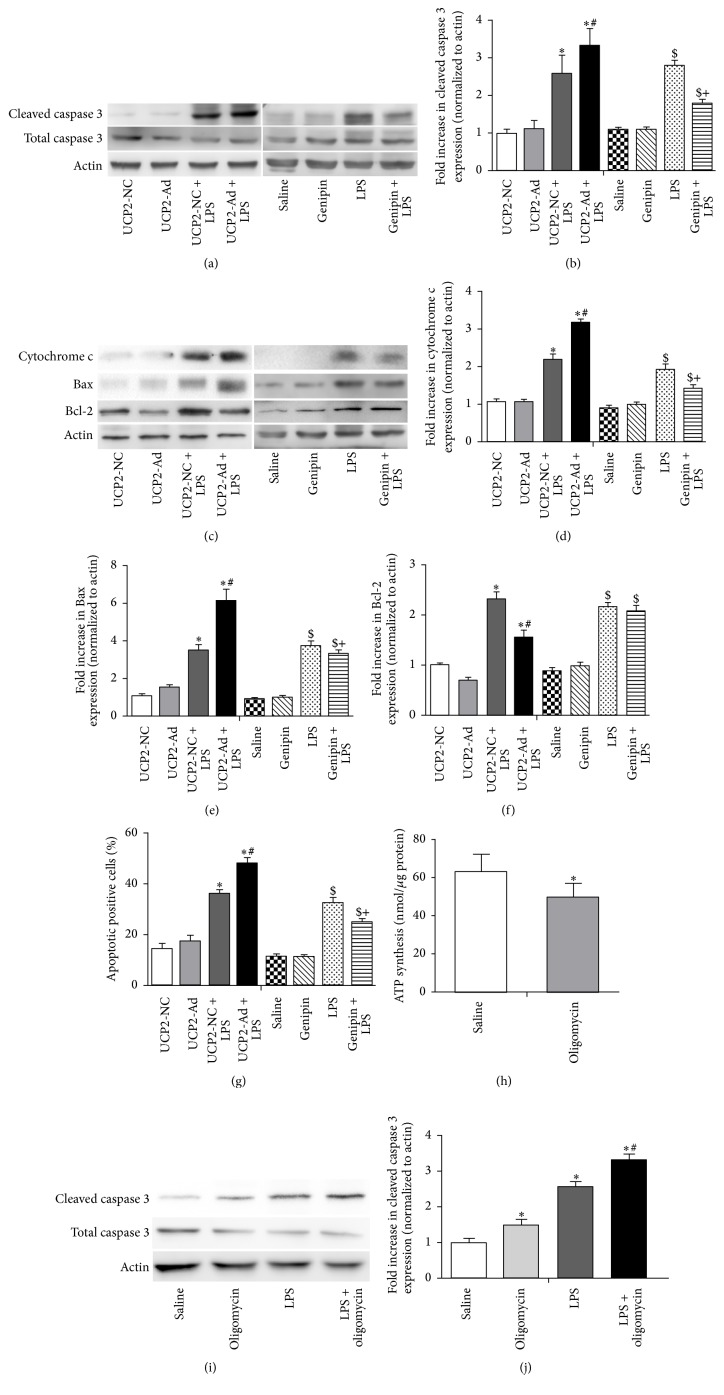
UCP2 enhanced DNA damage and increased caspase-3 cleavage, cytochrome c release, and proapoptotic Bax protein expression but reduced Bcl-2 expression. (a) Western blot showed the expression of cleaved and total caspase-3 from lung tissues of mice in each group. (c) Western blot analysis detected the expression of Bcl-2, Bax, and cytochrome c protein in each group. (b, d–f) Densitometric analysis of these proteins immunoreactive bands is shown as fold difference normalized to actin expression. (g) The percentage of apoptotic positive cells was detected by the TUNEL assay. (h) The mitochondrial ATP levels were measured by a luciferase-based assay after oligomycin (1 mg/kg) preinjection in mice. (i) Western blot analysis detected the expression of cleaved and total caspase-3. (j) Densitometric analysis of cleaved caspase-3 proteins immunoreactive bands is shown as fold difference normalized to actin expression. (^*∗*^ *P* < 0.05* versus* the UCP2-NC + saline group, ^#^ *P* < 0.05* versus* the UCP2-NC + LPS group, ^$^ *P* < 0.05* versus* the saline group, and ^+^ *P* < 0.05* versus* the LPS group). Values are presented as means ± SEM (*n* = 3).

Next, the effect of UCP2 overexpression on alveolar apoptosis in LPS-induced ALI was examined. The expression of total caspase-3 and cleaved caspase-3 was assessed by Western blot analysis (Figure 4(a)). The amount of the cleaved form of caspase-3 was significantly increased in lung tissue of UCP2-Ad + LPS mice compared to the UCP2-NC + LPS group (Figure 4(c)). Furthermore, the change of Bcl-2 family protein expression was assessed in lung tissue (Figure 4(c)). Whereas the level of antiapoptotic protein Bcl-2 was decreased, the amount of proapoptotic protein Bax was increased in the UCP2-Ad group compared to the UCP2-NC group after LPS treatment. In addition, release of cytochrome c from mitochondria was increased in lung tissue of UCP2-Ad mice after LPS treatment (Figures 4(c) and 4(d)). A TUNEL assay was performed to detect cell death in mouse lung tissues and the results revealed that the level of DNA damage was significantly increased in the UCP2-Ad group compared to the UCP2-NC group after LPS treatment (Figure 4(g)), suggesting that UCP2 further enhanced cell death after LPS treatment. In contrast, genipin inhibition of UCP2 reduced the LPS-induced increase of cleaved caspase-3 (Figures 4(a) and 4(b)), cytochrome c, and Bax (Figures 4(c)–4(f)) and finally protected alveolar epithelial against UCP2-induced cell death (Figure 4(g)). To reveal the role of UCP2 mediated ATP depletion in cell death, the ATP inhibitor oligomyin was used to decrease ATP in the lungs of mice (Figure 4(h)), which increased the expression of cleaved caspase-3 in lungs after LPS treatment (Figures 4(i) and 4(j)). Collectively, these experiments indicate that UCP2 promoted LPS-induced apoptosis by ATP depletion and shifting the balance between proapoptotic Bax and antiapoptotic Bcl-2 protein.

### 3.5. UCP2-Induced Inflammation in LPS-Induced ALI in Mice Is Mediated by JNK and p38 MAPK Pathways

**Figure 5:**
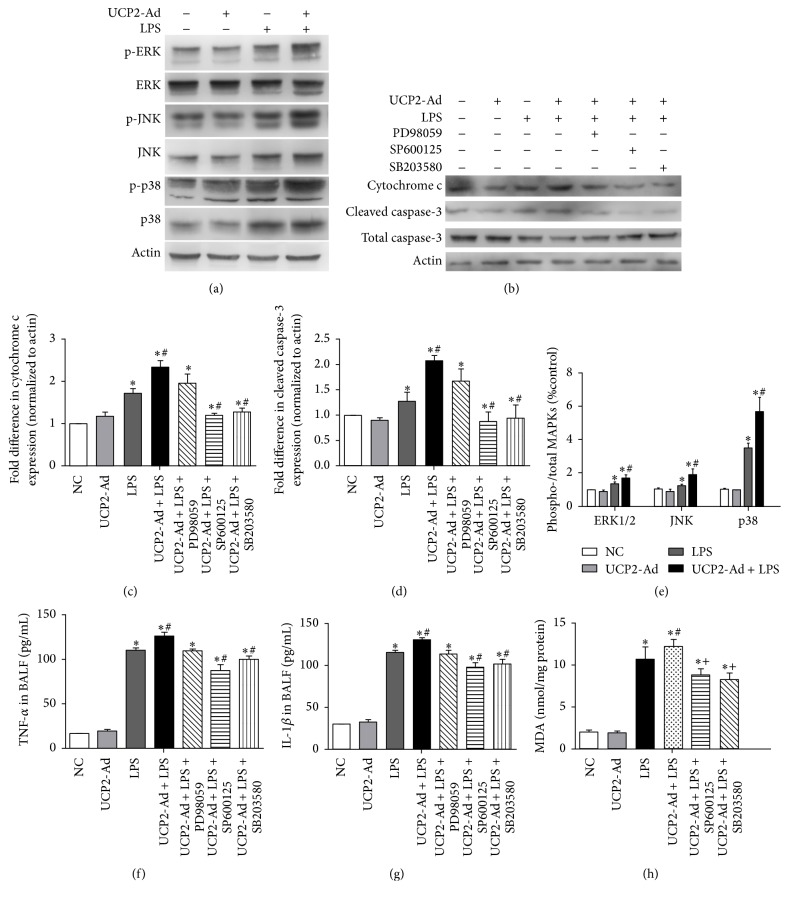
UCP2-induced inflammation in LPS-induced ALI is mediated by JNK and p38 MAPK pathway. (a) Western blot detected the expression of ERK, JNK, and p38 MAPKs and their phosphorylated protein in lung tissue after LPS-induced injury. (b) Western blot detected cytochrome c and caspase-3 expression after inhibition of UCP2-induced activation of MAPK signaling pathways by the inhibitors of specific MAPK signaling pathways, respectively. (c-d) Quantification of cytochrome c and caspase-3 immunoreactivity using Western blot analysis. (e) Quantification of the phosphorylated protein p-ERK, p-JNK, and p-p38 shown in graphs using densitometric analysis. (f) The levels of TNF-*α* in BALF were measured by ELISA. (g) The levels of IL-1*β* in BALF were measured by ELISA. (h) The levels of ROS in lung were detected by MDA assay (^*∗*^ *P* < 0.05* versus* NC group, ^#^ *P* < 0.05* versus* LPS group, and ^+^ *P* < 0.05* versus* UCP2-Ad + LPS group). Values are presented as means ± SEM (*n* = 3).

LPS-induced cell apoptosis depends on MAPKs activation [28]. Thus, to examine the mechanisms by which UCP2 overexpression in the LPS-induced ALI mice enhances apoptosis, the role of MAPKs in LPS-induced apoptosis in lung was investigated. The quantity of ERK, p38 MAPK (p38), JNK, and their phosphorylated forms were detected by Western blot analysis (Figure 5(a)). The data showed that UCP2 overexpression further enhanced phosphorylation of ERK, p38, and JNK protein in response to LPS (Figure 5(e)).

In order to confirm the involvement of MAPK signaling and to determine the effect on UCP2-induced apoptosis and inflammatory factor release, ERK inhibitor (PD98059), JNK inhibitor (SP600125), and p38 MAPK inhibitor (SB203580) were used to block MAPK signaling. Inhibition of MAPK activity by JNK or p38 MAPK inhibitors, but not the ERK inhibitor, revealed involvement of JNK and p38, but not ERK in the modulation of UCP2-induced apoptosis in ALI mice (Figures 5(b)–5(d)). Similarly, MAPK inhibitors SP600125 and SB203580, but not PD98059, significantly reduced TNF-*α* (Figure 5(f)) and IL-1*β* (Figure 5(g)) in BALF of UCP2-Ad + LPS mice compared to UCP2-NC + LPS mice, demonstrating that enhanced UCP2 expression increased LPS-induced lung inflammation* via* the JNK and p38 MAPK pathways. The MDA assay showed that the MAPK inhibitors SP600125 and SB203580 reduced the level of ROS after LPS treatment (Figure 5(h)), suggesting UCP2-mediated ROS increase via the JNK and P38 pathways.

**Figure 6:**
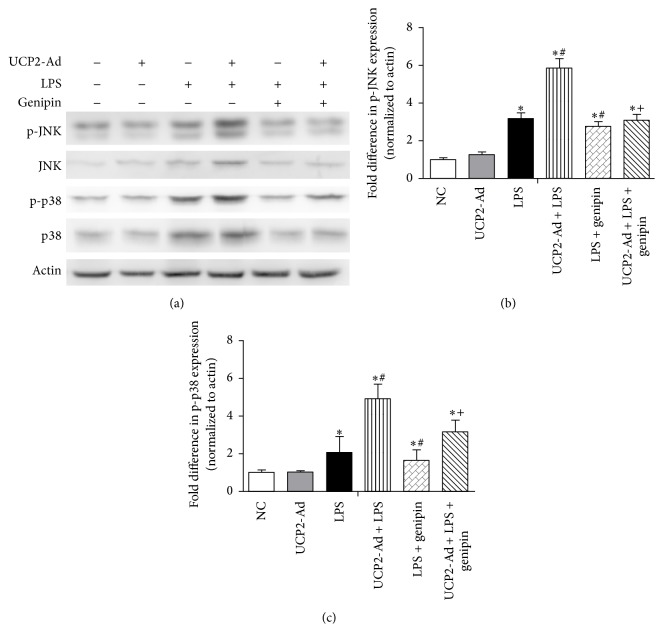
Genipin attenuated LPS-induced inflammatory lung injury via JNK and p38 MAPK pathway. (a) Western blot analysis shows the role of genipin in the activation of JNK and p38 in lung tissue after ALI. (b) Quantification of p-JNK immunoreactivity using Western blot analysis. (c) Quantification of p-p38 immunoreactivity using Western blot analysis. (^*∗*^ *P* < 0.05* versus* NC group, ^#^ *P* < 0.05* versus* LPS group, and ^+^ *P* < 0.05* versus* UCP2-Ad + LPS group). Values are presented as means ± SEM (*n* = 3).

Moreover, the UCP inhibitor genipin reduced phosphorylation of p38 and JNK in LPS-induced ALI mice (Figures 6(a)–6(c)), which was consistent with the findings that UCP2 expression promotes LPS-induced ALI in mice and specific inhibition of UCP2 function alleviates inflammatory lung injury.

## 4. Discussion

Lung inflammation and pulmonary epithelial apoptosis play critical roles in the progression of ALI. UCP2, an anionic proton transporter, participates in inflammation. However, whether or not UCP2 plays an important role in LPS-induced lung injury is still unclear. In the current study, we demonstrated the effect of UCP2 in LPS-induced inflammatory lung injury. LPS administration induced inflammatory lung injury, including elevation of inflammatory cell infiltration and inflammatory factor release and activation of MAPK signaling pathways and alveolar epithelial cell death. Overexpression of UCP2 in lung tissues with recombinant adenovirus significantly activated the MAPKs pathway, elicited inflammatory cell infiltration, and elevated pulmonary permeability. Moreover, UCP2-induced ATP depletion, decreased mitochondrial membrane potential and increased caspase-3 activation, Bax and cytochrome c protein expression, and DNA fragmentation in LPS-induced ALI, while UCP2 inhibition with genipin attenuated LPS-induced alveolar epithelial cell death and inflammation. These findings suggest that UCP2 increases the degree of LPS-induced lung injury by promoting an inflammatory response and increasing pulmonary epithelial apoptosis in mice.

Since UCP2-knockout and overexpressing mice are not available, we used recombinant adenovirus and the UCP2 inhibitor, genipin, to modulate UCP2 expression to investigate the functional role of UCP2 in LPS-induced ALI. We performed a UCP2-Ad gradient dose experiment and determined that 5 × 10^8^ pfu/mouse UCP2-Ad resulted in optimal UCP2 overexpression without triggering lung injury. In addition, compared to the UCP2-NC + LPS group, UCP2 expression in the UCP2-Ad + LPS group was increased, indicating successful adenovirus gene delivery to the lungs of mice* via* intranasal instillation. Genipin has been widely used to specifically inhibit UCP2 expression [27]. Our results revealed that UCP2 expression was inhibited by genipin following UCP2-Ad or LPS treatment in mouse lungs. UCP2 may exert a protective effect in myocardial ischemia [29] and cerebral ischemia [12], while an earlier study indicated that UCP2 accelerates disease progression in amyotrophic lateral sclerosis (ALS) [25]. Some studies also showed that UCP2 overexpression may be deleterious in acute liver injury [14] and pancreatitis [30]. Our study revealed that UCP2-Ad treatment further lowered the survival rate in mice after LPS treatment, whereas genipin increased the survival rate. One possible explanation may be that UCP2-Ad pretreatment aggravated inflammatory cell infiltration, which led to the release of inflammatory factors TNF-*α* and IL-1*β*, into the lung tissue, and increased pulmonary edema and alveolar epithelial cell death rates. We clearly demonstrated that UCP2 increased the degree of LPS-induced lung injury by promoting an inflammatory response and increasing pulmonary epithelial apoptosis and that UCP2 downregulation may be beneficial.

The physiological function of UCP2 is the subject of intense debate. Some studies suggested that UCP2 is an antioxidant and scavenges ROS, thus stabilizing the inner mitochondrial membrane potential [31]. Despite UCP2's proposed protectant role in diminishing oxidative damage, the available* in vivo* data is still not convincing, and some results even indicate that UCPs are detrimental factors under conditions of oxidative stress [32]. Although UCP2 overexpression reduced ROS production in the endothelium of the aorta and the mesenteric artery in mice [33], proton leakage and ROS production were not significantly different in splenic mitochondria compared to control [34]. Therefore, the role of UCP2 in ROS generation and cell death appears to depend on tissue type and stimulus applied. Our results showed that UCP2-mediated ROS increase via JNK and P38 pathways, suggesting the absence of any protective effect by UCP2, as well as the rather increased ROS production following LPS administration, indicating UCP2 enhanced mitochondrial dysfunction in oxidative damage following LPS-induced ALI rather than playing a protective role.

Concomitant with ROS production, UCP2 overexpression also led to mitochondrial dysfunction in mouse lungs. The study by Islam et al. [35] showed that UCP2 overexpression enhanced mitochondrial function and improved energy homeostasis. In contrast, Peixoto et al. [25] suggested that UCP2 enhances mitochondrial dysfunction. Our data demonstrated that the potential mechanism by which UCP2 triggers the apoptosis machinery includes changes in energy metabolism and activation of mitochondrial signaling pathways. The ATPase inhibitor oligomycin could promote LPS-induced cell death. The reduction of ATP synthesis may explain how UCP2 contributed to LPS-induced enhancement of apoptosis. These results are similar to prior studies, which indicated that the reduction of ATP synthesis induces JNK and p38 MAPK activation [36]. Our data showed that UCP2 enhanced MAPKs activation and reduced Bcl-2 expression, which can reduce Bax-stimulated apoptotic events. Additionally, we also showed that UCP2 enhanced Bax expression, which is consistent with previous reports suggesting that activated MAPK signaling pathways regulate Bax translocation from the cytoplasm to mitochondria causing apoptosis [37]. Moreover, consistent with what has already been reported in a mouse model of ALS [35], our findings suggest that UCP2 overexpression reduced mitochondrial membrane potential, which may increase cytochrome c release. Finally, activation of caspase-3 and increased DNA fragmentation were observed. Therefore, UCP2 appears to exacerbate LPS-induced apoptosis* via* an intrinsic ATP-MAPKs-Bax/Bcl-2-mitochondrial-Cyt c-caspase signaling pathway.

MAPK pathways (ERK, JNK, and P38) play a critical role in regulating the release of inflammatory factors and cell apoptosis and participate in LPS-induced ALI [38]. JNK and P38 regulate inflammatory factor TNF-*α* and IL-1*β* release [39] and increase pulmonary epithelial apoptosis [40]. In the present study, we found that UCP2 significantly increased MAPK activation. To determine which MAPK pathway is involved in the enhanced UCP2-induced inflammatory factor release and apoptosis, MAPK inhibitors were used to specifically block distinct MAPK pathways. These results revealed an involvement of JNK and p38, but not ERK, in the modulation of UCP2-induced inflammatory factor release and apoptosis in mouse ALI.

Based on these findings, we next downregulated UCP2 expression to provide new insights into the potential effects of UCP2 inhibitor pretreatment on LPS-induced lung injury* via* the JNK and p38 MAPK pathways. Our results showed that UCP2 expression was upregulated in lungs of mice with ALI. Our finding is in line with a study by Moon et al., showing that UCP2 promoted mortality in experimental sepsis and that its expression was increased in patients with systemic inflammation and infection [17]. Increased expression of UCP2 may enhance inflammatory responses in ALI and sepsis. In this regard, the UCP2 inhibitor, genipin, downregulated endogenous UCP2 expression following LPS-induced ALI in mice. Thus, genipin is a useful tool for further evaluation of the function of UCP2 in ALI. The significant reduction in inflammatory response in lung tissue and marked reduction of cell apoptosis by genipin suggested that UCP2 inhibition reduces lung injury by attenuating inflammatory responses and apoptosis following LPS treatment. Moreover, the significantly reduced lung edema, decreased infiltration of inflammatory cells, reduced protein concentration of BALF, and decreased release of inflammatory cytokines by genipin further confirmed its effect on the improvement of lung injury in mice with ALI.

In conclusion, the present study revealed that UCP2 expression exacerbated acute lung injury following LPS treatment. These results provided further evidence that treatment with recombinant adenovirus encoding UCP2* in vivo* enhanced mitochondrial dysfunction, suggesting that the dissociation between mitochondrial dysfunction and ROS signaling related to UCP2 is much more complex than we currently understand and that the role of UCP2 in lung diseases and other physiological and pathological conditions needs to be further investigated. A better understanding of the role of UCP2 in inflammation may be promising for developing new treatments for lung diseases in the future.
